# The risk of perinatal and cardiometabolic complications in pregnancies conceived by medically assisted reproduction

**DOI:** 10.1007/s10815-024-03025-9

**Published:** 2024-01-20

**Authors:** Dovile Vilda, Elizabeth F. Sutton, Venkata Sai Sahithi Kothamasu, Paul R. Clisham, Cecilia T. Gambala, Emily W. Harville

**Affiliations:** 1 Department of Social, Behavioral, and Population Sciences, Mary Amelia Center for Women’s Health Equity Research, Tulane University School of Public Health and Tropical Medicine, New Orleans, LA USA; 2 Woman’s Hospital, Baton Rouge, LA USA; 3 Tulane University School of Medicine, New Orleans, LA USA; 4 Department of Obstetrics and Gynecology, Tulane University School of Medicine, New Orleans, LA USA; 5 Department of Epidemiology, Tulane University School of Public Health and Tropical Medicine, New Orleans, LA USA

**Keywords:** Medically assisted reproduction, Electronic medical records, Perinatal, Cardiometabolic complications, Propensity score matching

## Abstract

**Purpose:**

To examine the impact of medically assisted fertility treatments on the risk of developing perinatal and cardiometabolic complications during pregnancy and in-hospital deliveries.

**Methods:**

We conducted a retrospective cohort study using medical health records of deliveries occurring in 2016–2022 at a women’s specialty hospital in a southern state of the Unites States (US). Pregnancies achieved using medically assisted reproductive (MAR) techniques were compared with unassisted pregnancies using propensity score matching (PSM), based on demographic, preexisting health, and reproductive factors. Study outcomes included cesarean delivery, gestational diabetes mellitus (GDM), hypertensive disorders of pregnancy (HDP), delivery complications, and postpartum readmission. We used Poisson regression with robust standard errors to generate risk ratios (RRs) and 95% confidence intervals (CIs) for all study outcomes.

**Results:**

Among 57,354 deliveries, 586 (1.02%) pregnancies were achieved using MAR and 56,768 (98.98%) were unassisted (“non-MAR”). Compared to the non-MAR group, MAR pregnancies had significantly higher prevalence of all study outcomes, including GDM (15.9% vs. 11.2%, *p* < 0.001), HDP (28.2% vs. 21.1%, *p* < 0.001), cesarean delivery (56.1% vs. 34.6%, *p* < 0.001), delivery complications (10.9% vs. 6.8%, *p* = 0.03), and postpartum readmission (4.3% vs. 2.7%, *p* = 0.02). In a PSM sample of 584 MAR and 1,727 unassisted pregnancies, MAR was associated with an increased risk of cesarean delivery (RR = 1.11, 95% CI = 1.01–1.22); whereas IVF was associated with an increased risk of cesarean delivery (RR = 1.15, 95% CI = 1.03–1.28) and delivery complications (RR = 1.44, 95% CI = 1.04–2.01).

**Conclusions:**

Women who conceived with MAR were at increased risk of cesarean deliveries, and those who conceived with IVF were additionally at risk of delivery complications.

**Supplementary Information:**

The online version contains supplementary material available at 10.1007/s10815-024-03025-9.

## Introduction

Infertility (i.e., the inability to conceive within 12 months of trying) is a public health issue affecting up to 15% reproductive-age individuals in the US [1, 2] that is associated with chronic health conditions including greater body mass index (BMI), diabetes, and cardiovascular disease (CVD) risks [3–5]. Medically assisted reproduction (MAR) techniques, which encompass assisted reproductive technologies (ART) such as in vitro fertilization (IVF) and intracytoplasmic sperm injection (ICSI), and non-ART procedures such as ovulation induction (OI) and intrauterine insemination (IUI), emerged as safe and feasible options for conceiving for many people. While during the past two decades the use of MAR has steadily increased in the US and around the globe [1, 6], growing evidence has documented higher risks for adverse obstetric and perinatal outcomes among MAR-treated populations [1, 7–10]. Numerous studies have established that pregnancies conceived with MAR are more likely to result in higher incidence of preterm labor, preterm delivery, and low birthweight compared with spontaneously conceived births [8–13]. This has been commonly attributed to the MAR-treated populations being older, having more preexisting health conditions, experiencing an increased risk for multiple gestation pregnancies, and thus being at higher risk for adverse pregnancy and birth outcomes [1, 10].

While infertile women experience a higher level of cardiometabolic and cardiovascular disease risk factors [14, 15], the evidence on the impact of fertility treatment on aggravating the risk for cardiometabolic and cardiovascular complications during pregnancy and in later life is less clear [3, 10, 16, 17]. While one review and meta-analysis concluded that infertility treatment may not increase the risk for longer term cardiovascular events in MAR-treated individuals [16], more recent studies have documented the adverse effects of fertility treatment on hypertensive disorders of pregnancy (HDP) and severe maternal morbidity (SMM) [17–20]. In addition, the presence and severity of preexisting cardiometabolic and cardiovascular risk factors have been shown to predict pregnancy complications and maternal and neonatal morbidity [4]. Prior studies have not established the extent to which increased cardiometabolic and cardiovascular risks and complications during pregnancy and the early postpartum period are related to MAR treatment, the preexisting health conditions that underly infertility diagnosis, or their combination.

Several potential mechanisms may explain how MAR could increase the risk for cardiometabolic complications during pregnancy and childbirth. First, chronic health conditions and cardiometabolic risk factors, such as advanced maternal age, diabetes, hypertension, and obesity, are known to contribute to infertility and are more prevalent among patients undergoing MAR [2, 5, 10, 21]. These fertility related conditions, including polycystic ovary syndrome (PCOS) and endometriosis, are also linked to a higher risk of cardiometabolic and pregnancy complications [5]. Furthermore, the use of MAR may pose additional cardiac and metabolic stress during pregnancy due to the use of drug therapies, ovarian stimulation, and hormonal medications that stimulate the ovulation which in turn leads to marked fluctuations in endogenous estrogen, as well as inherent risks due to multiple gestation [5]. Recent evidence also suggests that MAR-seeking individuals may have accelerated biological aging which may also affect their risk for chronic diseases and cardiometabolic complications [22].

The aim of the current study was to examine the associations between the use of MAR and risk of developing perinatal and cardiometabolic complications during pregnancy and childbirth in a large, contemporaneous, diverse population. In so doing, we adjusted for preexisting cardiometabolic risk factors and additionally matched the two groups (MAR-treated and non-MAR) on a propensity score, generated based on demographic, preexisting health, and reproductive factors. Building on growing evidence [2, 21] and the mechanisms discussed above, we hypothesized that the use of MAR will be associated with increased risk of perinatal and cardiometabolic complications during pregnancy and childbirth.

## Methods

### Study design and data sources

A retrospective cohort study was conducted using electronic medical records of all deliveries at Woman’s Hospital, Baton Rouge, Louisiana, from January 2016 through December 2022. Woman’s Hospital is a tertiary obstetric hospital in South-central Louisiana, accounting for approximately 15% of all births in Louisiana annually [23]. Inclusion criteria for the study limited the sample to all in-hospital deliveries (live and stillbirths, singletons and multiple gestations) to birthing persons over 18 years old during the study period (*N* = 57,354). Ethical approval for the study was obtained from the Woman’s Hospital Foundation Institutional Review Board.

### Measures

Based on conception method indicated on medical records, deliveries were categorized into those achieved using MAR (including IVF and IUI) and unassisted pregnancies (non-MAR). Primary outcomes included cesarean delivery, diagnosis of any hypertensive disorder of pregnancy (HDP), and gestational diabetes mellitus (GDM). Secondary outcomes included delivery complications (defined as a range of medical issues or problems occurring during the process of delivery, including one or more injuries such as uterine rapture, uterine atony, placental abruption, and immediate postpartum hemorrhage (see Supplement for ICD-10 codes)) and postpartum readmission. All outcome variables were dichotomous.

Maternal information considered as potential confounders included maternal age at delivery (18–24, 25–29, 30–34, 35–39, ≥ 40 years old), race/ethnicity (Asian, Hispanic, non-Hispanic (NH) Black, NH White, another), educational attainment (less than high school, high school, some college, college degree, advanced degree, missing/unknown), marital status (married vs. other), insurance (private, Medicaid/Medicare, other), parity (0, 1, 2, ≥ 3), history of smoking (ever smoker vs. non-smoker), drinking during pregnancy (non-drinker vs. other), illicit drug use during pregnancy (yes/no). The pre-delivery body mass index (BMI) was calculated using the weight and height recorded upon admissions for delivery, and obesity was dichotomized into those ≥ 30 kg/m^2^ vs. <30 kg/m^2^. Preexisting health conditions included chronic hypertension, diabetes mellitus, pulmonary disease, a diagnosis of mood and anxiety disorder, and history of sexually transmitted infections (STIs), whereas characteristics of index pregnancy included multiple gestation and breech fetal presentation at delivery, all retrieved from medical records.

### Statistical analysis

First, we assessed the frequency distribution of baseline characteristics of patients by conception categories (MAR vs. non-MAR) using t-tests for continuous variables and chi-square tests for categorical variables. Second, because MAR-seeking individuals may systematically differ from non-MAR individuals on numerous factors contributing to outcome measures, we used a propensity score matching (PSM) approach to identify individuals who were most similar to the MAR-treated group. The PSM analysis is a statistical tool used to minimize selection bias and confounding factors in observational studies [24] and thus estimating a propensity score for MAR treatment and matching based on the score helps determine whether MAR has a causal effect on perinatal and cardiometabolic complications. To address potential confounding by indication, we matched 3 unassisted pregnancies to each MAR pregnancy using a propensity score which was calculated based on maternal sociodemographic (age at delivery, race/ethnicity, educational attainment, marital status, insurance), preexisting health conditions (obesity, substance use, chronic hypertension, diabetes mellitus, pulmonary disease, mood and anxiety disorders, history of STIs), and index pregnancy characteristics (parity, fetal presentation). The propensity score was generated by including these baseline characteristics in a logistic regression model; pregnancies were matched without replacement using the greedy method with a caliper width score of 0.25 standard deviations [24]. Baseline characteristics and study outcomes in the PSM sample were also assessed.

Third, we performed Poisson regression analysis with robust standard errors to generate risk ratios (RRs) and 95% confidence intervals (CIs) for all study outcomes for both matched and unmatched samples. Model 1 examined unadjusted (crude) associations between MAR and study outcomes in the original sample, while Model 2 adjusted for all maternal characteristics indicated above (i.e., sociodemographic, preexisting health conditions, and index pregnancy characteristics). In assessing the risk for HDP and GDM, we excluded those with preexisting or chronic hypertension (i.e., hypertension identified before the 20th week of pregnancy) and preexisting type 1 or 2 diabetes, respectively. Model 3 examined the associations between MAR and study outcomes in the PSM sample.

We also conducted several sensitivity analyses. In the first one, we limited the sample to singleton nulliparous deliveries to determine if study outcomes were influenced by multiple births and/or those with previous deliveries in this study cohort. Secondly, we examined whether more invasive fertility treatment (i.e., IVF) is associated with higher risk for adverse cardiometabolic and perinatal outcomes. Finally, we additionally adjusted for the history of cesarean deliveries in examining the association between the MAR treatment and cesarean delivery of the index pregnancy. All statistical analyses were performed in SAS v.9.3 (SAS Institute Inc, Cary, NC); *P* values < 0.05 (two-sided) were considered statistically significant.

## Results

### Sample characteristics

A comparison of baseline characteristics and perinatal and cardiometabolic outcomes between MAR and non-MAR groups is shown in Table 1. Among 57,354 deliveries in the original sample, 584 (1.02%) pregnancies were achieved using MAR and 56,768 (98.98%) were unassisted conceptions. Among the MAR group, 476 (81.2%) used IVF treatment and the remaining 110 (18.8%) used IUI treatment. Compared to their peers, women who conceived with MAR were more likely to be over 35 (59.7% vs. 23.5%), non-Hispanic White (77.1% vs. 50.2%), with a college or higher degree (72.7% vs. 36.9%), married (94.9% vs. 51.1%), and privately insured (94.0% vs. 52.4%). Compared to MAR group, non-MAR patients were more likely to be current or former smokers (10.6% vs. 5.9%, *p* < 0.001), use drugs (8.0% vs. 0.3%, *p* < 0.001), had greater incidence of pulmonary disease (12.0% vs. 8.5%, *p* < 0.05), and lesser prevalence of chronic hypertension (11.4% vs. 8.2%, *p* < 0.05), and obesity (55.5% vs. 60.2%, *p* = 0.02). There were no significant differences in alcohol use during pregnancy, diabetes mellitus, mood and anxiety disorders, and the history of STIs. With respect to index pregnancy characteristics, a greater proportion of MAR patients was nulliparous (62.9% vs. 37.8%, *p* < 0.001), with multiple gestation (20.7% vs. 4.0%, *p* < 0.001), and breeched fetal presentation (14.9% vs. 5.4%, *p* < 0.001).

**Table 1:** Baseline characteristics of the study sample before and after propensity score matching

Characteristic	Unmatched sample (*N* = 57,354)			Propensity score-matched sample (*N* = 2,311)		
	*N* (%) of women with **no** fertility treatment (*N* = 56,768)	*N* (%) of women with fertility treatment (*N* = 586)	*P*-value of the difference	*N* (%) of women with **no** fertility treatment (*N* = 1,727)	*N* (%) of women with fertility treatment (*N* = 584)	*P*-value of the difference
**Year**						
*2016*	8525 (15.0)	81 (13.8)	*p* = 0.16	278 (16.1)	79 (13.5)	*p* < 0.01
*2017*	8204 (14.5)	77 (13.1)		275 (15.9)	77 (13.2)	
*2018*	8030 (14.2)	74 (12.6)		314 (18.2)	74 (12.7)	
*2019*	8070 (14.2)	79 (13.5)		228 (13.2)	79 (13.5)	
*2020*	8089 (14.3)	80 (13.7)		260 (15.1)	80 (13.7)	
*2021*	7944 (13.9)	103 (17.6)		185 (10.7)	103 (17.6)	
*2022*	7906 (13.9)	92 (15.7)		187 (10.8)	92 (15.8)	
***Maternal sociodemographic characteristics***						
**Age at index pregnancy (years)**						
Mean (SD)	28.8 (5.5)	34.3 (5.1)	*p* < 0.001	34.4 (4.5)	34.3 (4.7)	*p* = 0.63
*18–24*	13,474 (23.7)	11 (1.9)	*p* < 0.001	23 (1.3)	11 (1.9)	*p* = 0.85
*25–29*	17,791 (31.3)	76 (12.9)		211 (12.2)	76 (13.0)	
*30–34*	16,352 (28.8)	213 (36.4)		638 (36.9)	213 (36.5)	
*35–39*	7614 (13.4)	203 (34.6)		622 (36.0)	203 (34.8)	
*> 40*	1537 (2.7)	83 (14.2)		233 (13.5)	81 (13.9)	
**Maternal race/ethnicity**						
*Asian*	1249 (2.2)	30 (5.2)	*p* < 0.001	62 (3.6)	30 (5.1)	*p* = 0.55
*Black*	21,115 (37.2)	76 (12.9)		239 (13.8)	76 (13.0)	
*Hispanic*	4781 (6.7)	25 (4.3)		71 (4.1)	25 (4.3)	
*White*	28,482 (50.2)	452 (77.1)		1344 (77.8)	450 (77.1)	
*Another*	1139 (3.7)	3 (0.5)		11 (0.6)	3 (0.5)	
**Marital status**						
*Married*	29,029 (51.1)	556 (94.9)	*p* < 0.001	1637 (94.8)	554 (94.9)	*p* = 0.94
*Other*	27,739 (48.9)	30 (5.1)		90 (5.2%)	30 (5.1)	
**Education**						
*Less than high school degree*	5546 (9.8)	10 (1.7)	*p* < 0.001	26 (1.5)	10 (1.7)	*p* = 0.98
*High school degree*	12,653 (22.3)	32 (5.5)		104 (6.0)	32 (5.5)	
*Some college*	11,482 (20.2)	75 (12.8)		230 (13.3)	73 (12.5)	
*College graduate*	15,944 (28.1)	261 (44.5)		772 (44.7)	261 (44.7)	
*Advanced degree*	5003 (8.8)	165 (28.2)		476 (27.6)	165 (28.3)	
*Missing data*	6140 (10.8)	43 (7.3)		119 (6.9)	43 (7.4)	
**Insurance**						
*Private*	29,718 (52.4)	551 (94.0)	*p* < 0.001	1640 (94.9)	549 (94.0)	*p* = 0.59
*Medicaid or Medicare*	26,772 (47.2)	29 (5.0)		75 (4.3)	29 (4.9)	
*Other*	278 (0.5)	6 (1.0)		12 (0.7)	6 (1.0)	
***Maternal health status***						
Smoking	6008 (10.6)	35 (5.9)	*p* < 0.001	111 (6.4)	35 (6.0)	*p* = 0.71
Alcohol use	3183 (5.6)	43 (7.3)	*p* = 0.07	102 (5.9)	43 (7.4)	*p* = 0.21
Illicit drug use	4553 (8.0)	2 (0.3)	*p* < 0.001	9 (0.5)	2 (0.3)	*p* = 0.59
History of STIs	15,390 (27.1)	106 (18.1)	*p* < 0.001	267 (15.5)	106 (18.2)	*p* = 0.13
Chronic hypertension	4661 (8.2)	67 (11.4)	*p* < 0.01	174 (10.1)	67 (11.5)	*p* = 0.34
Diabetes mellitus	748 (1.3)	9 (1.5)	*p* = 0.65	15 (0.9)	9 (1.5)	*p* = 0.16
Pulmonary disease	6833 (12.0)	50 (8.5)	*p* < 0.01	155 (8.9)	50 (8.6)	*p* = 0.76
Mood and anxiety disorder	16,405 (28.9)	191 (32.6)	*p* = 0.05	584 (33.8)	189 (32.4)	*p* = 0.52
Obesity	34,188 (60.2)	325 (55.5)	*p* = 0.02	974 (56.4)	323 (55.3)	*p* = 0.66
**Pregnancy characteristics**						
Parity						
*0*	21,163 (37.8)	364 (62.9)	*p* < 0.001	1098 (64.0)	362 (62.7)	*p* = 0.95
*1*	18,051 (32.2)	170 (29.4)		486 (28.3)	170 (29.5)	
*2*	10,054 (17.9)	29 (5.0)		83 (4.8)	29 (5.0)	
*≥ 3*	6795 (12.1)	16 (2.8)		48 (2.8)	16 (2.8)	
Fertility treatment						
*IUI*	-	110 (18.8)	-	-	110 (18.8)	-
*IVF*	-	476 (81.2)		-	474 (81.2)	
Multiple gestation	2280 (4.0)	121 (20.7)	*p* < 0.001	271 (15.7)	119 (20.4)	*p* = 0.01
Breech presentation	3081 (5.4)	86 (14.9)	*p* < 0.001	187 (10.8)	86 (14.7)	*p* = 0.01
**Study outcomes**						
*Cesarean delivery*	19,665 (34.6)	329 (56.1)	*p* < 0.001	823 (47.7)	327 (55.9)	*p* < 0.001
*GDM*	6370 (11.2)	93 (15.9)	*p* < 0.001	290 (16.8)	93 (15.9)	*p* = 0.63
*HDP*	11,976 (21.1)	165 (28.2)	*p* < 0.001	410 (23.7)	165 (28.3)	*p* = 0.03
*Delivery complications*	3880 (6.8)	64 (10.9)	*p* = 0.03	131 (7.6)	62 (10.6)	*p* = 0.02
*Postpartum readmission*	1540 (2.7)	25 (4.3)	*p* = 0.02	58 (3.4)	25 (4.3)	*p* = 0.30

GDM, gestational diabetes mellitus; HDP, hypertensive disorders of pregnancy; SD, standard deviation; IUI, intrauterine insemination; IVF, in vitro fertilization

Delivery complications include uterine rapture, uterine atony, placental abruption, and immediate postpartum hemorrhage

MAR pregnancies had a significantly higher prevalence of all study outcomes compared to non-MAR group, including GDM (15.9% vs. 11.2%, *p* < 0.001), HDP (28.2% vs. 21.1%, *p* < 0.001), cesarean delivery (56.1% vs. 34.6%, *p* < 0.001), delivery complications (10.9% vs. 6.8%, *p* = 0.03), and postpartum readmission (4.3% vs. 2.7%, *p* < 0.05).

After propensity score matching, baseline characteristics of the MAR and non-MAR groups were not significantly different except for multiple gestation (20.4% vs. 15.7%, *p* = 0.01) and breech fetal presentation (14.7% vs. 10.8%, *p* = 0.01). As a result, we adjusted for multiple gestation and breech presentation in regression models with the PSM sample. A significantly higher prevalence of all study outcomes, except for GDM and postpartum readmission, also remained among the MAR patients in the PSM sample (Table 1).

### Associations between MAR and perinatal and cardio metabolic outcomes

Table 2 shows the relative risk associated with MAR treatment and study outcomes in the total eligible study sample, unadjusted (Model 1) and adjusted (Model 2) for baseline characteristics, and PSM sample (Model 3). Results from the crude models (Model 1) show that MAR group had increased risk for cesarean delivery (RR = 1.62; 95% CI = 1.49–1.76), GDM (RR = 1.41, 95% CI = 1.13–1.76), HDP (RR = 1.33, 95% CI = 1.15–1.55), and delivery complications (RR = 1.60, 95% CI = 1.22–2.09). No significant difference was observed between MAR and non-MAR groups in postpartum readmission (Table 2). After adjusting for maternal baseline characteristics (Model 2), increased risks in the MAR group remained for cesarean deliveries (aRR = 1.23, 95% CI = 1.13–1.34) and delivery complications (aRR = 1.46, 95% CI = 1.11–1.93).

**Table 2:** Pregnancy and delivery outcomes for MAR-conceived and unassisted pregnancies: unmatched (*N* = 57,354) and propensity-score matched (*N* = 2,311) samples

	Unmatched sample		Propensity score-matched sample
	Model 1 Crude RR (95% CI)	Model 2 Adjusted RR (95% CI)	Model 3 PSM RR (95% CI)
*Cesarean delivery*	1.62 (1.49–1.76)***	1.23 (1.13–1.34)***	1.13 (1.03–1.42)**
*Gestational diabetes mellitus*	1.41 (1.13–1.76)**	1.00 (0.81–1.25)	0.92 (0.72–1.17)
*Hypertensive disorders of pregnancy*	1.33 (1.15–1.55)***	1.07 (0.94–1.22)	1.13 (0.95–1.35)
*Delivery complications*	1.60 (1.22–2.09)***	1.46 (1.11–1.93)**	1.35 (0.97–1.87)
*Postpartum readmission*	1.57 (0.95–2.60)	1.25 (0.77–2.04)	1.21 (0.70–2.09)

Model 2 adjusted for maternal sociodemographic (age at delivery, race/ethnicity, educational attainment, marital status, insurance), preexisting health conditions (obesity, substance use, chronic hypertension, diabetes mellitus, pulmonary disease, mood and anxiety disorders, history of sexually transmitted infections (STI)), and index pregnancy characteristics (parity, multiple gestation, fetal presentation)

*p < 0.05; **p < 0.01; ***p < 0.001; Results in bold indicate a statistically significant result

MAR, medically assisted reproduction; PSM, propensity score matched sample; RR, Risk ratio

Delivery complications include uterine rapture, uterine atony, placental abruption, and immediate postpartum hemorrhage

In a PSM sample of 584 MAR and 1,727 unassisted pregnancies, MAR was associated with increased risk of cesarean delivery (RR = 1.11, 95% CI = 1.01–1.22) but not other outcomes included in the study (Table 2).

### Sensitivity analyses

Findings from the sensitivity analysis, in which we limited the sample to singleton nulliparous births (*N* = 20,650), were mostly consistent with the main findings (Table 3). In a fully adjusted model (Model 2), MAR was associated with an increased risk of cesarean deliveries (aRR = 1.25, 95% CI = 1.11–1.42). Additionally, in a PSM sample of 291 MAR and 869 unassisted singleton and nulliparous pregnancies, MAR was associated with an increased risk of cesarean delivery (RR = 1.20, 95% CI = 1.05–1.37) and delivery complications (RR = 1.53, 95% CI = 1.09–2.14).

**Table 3:** Pregnancy and delivery outcomes for MAR-conceived and unassisted singleton and nulliparous pregnancies: unmatched (*N* = 20,650) and propensity-score matched (*N* = 1,160, MAR *n* = 291, non-MAR *n* = 869) samples

	Unmatched sample (*N* = 20,650)		Propensity score-matched sample (*N* = 1,160)
	Model 1 Crude RR (95% CI)	Model 2 Adjusted RR (95% CI)	Model 3 PSM RR (95% CI)
*Cesarean delivery*	1.56 (1.38–1.76)***	1.25 (1.11–1.42)***	1.17 (1.04–1.31)**
*Gestational diabetes mellitus*	1.40 (1.05–1.87)*	0.92 (0.69–1.23)	0.88 (0.68–1.15)
*Hypertensive disorders of pregnancy*	1.21 (1.01–1.44)*	1.15 (0.99–1.33)	1.16 (0.96–1.39)
*Delivery complications*	1.27 (0.90–1.77)	1.38 (0.98–1.94)	1.53 (1.09–2.14)**
*Postpartum readmission*	0.62 (0.26–1.49)	0.56 (0.23–1.35)	0.56 (0.29–1.10)

Model 2 adjusted for maternal sociodemographic (age at delivery, race/ethnicity, educational attainment, marital status, insurance), preexisting health conditions (obesity, substance use, chronic hypertension, diabetes mellitus, pulmonary disease, mood and anxiety disorders, history of sexually transmitted infections (STI)), and index pregnancy characteristics (parity, fetal presentation)

*p < 0.05; **p < 0.01; ***p < 0.001; Results in bold indicate a statistically significant result

MAR, medically assisted reproduction; PSM, propensity score matched sample; RR, Risk ratio

Delivery complications include uterine rapture, uterine atony, placental abruption, and immediate postpartum hemorrhage

Table 4 shows results from sensitivity analysis on the impact of IVF treatment on study outcomes, which remains consistent with the results from the main models. In fully adjusted models (Model 2), IVF-treated group had increased risk of cesarean delivery (aRR = 1.22; 95% CI = 1.11–1.35) and delivery complications (aRR = 1.77, 95% CI = 1.34–2.35). In a PSM sample of 472 IVF and 1,388 unassisted singleton pregnancies, IVF was associated with an increased risk of cesarean delivery (RR = 1.15, 95% CI = 1.03; 1.28) and delivery complications (RR = 1.44, 95% CI = 1.04–2.01).

**Table 4:** Pregnancy and delivery outcomes for IVF and unassisted pregnancies: unmatched (*N* = 57,354) and propensity-score matched (*N* = 1,867, IVF *n* = 472, non-IVF *n* = 1,395) samples

	Unmatched sample (*N* = 57,354)		Propensity score-matched sample (*N* = 1,395)
	Model 1 Crude RR (95% CI)	Model 2 Adjusted RR (95% CI)	Model 3 PSM RR (95% CI)
*Cesarean delivery*	1.62 (1.47–1.78)***	1.22 (1.11–1.35)***	1.15 (1.03–1.28)*
*Gestational diabetes mellitus*	1.26 (0.98–1.62)	0.89 (0.69–1.14)	0.76 (0.58–1.01)
*Hypertensive disorders of pregnancy*	1.33 (1.12–1.57)***	1.10 (0.95–1.27)	1.13 (0.93–1.37)
*Delivery complications*	1.92 (1.46–2.51)***	1.77 (1.34–2.35)***	1.44 (1.04–2.01)**
*Postpartum readmission*	1.79 (1.07-3.00)*	1.42 (0.86–2.36)	1.33 (0.75–2.34)

Model 2 adjusted for maternal sociodemographic (age at delivery, race/ethnicity, educational attainment, marital status, insurance), preexisting health conditions (obesity, substance use, chronic hypertension, diabetes mellitus, pulmonary disease, mood and anxiety disorders, history of sexually transmitted infections (STI)), and index pregnancy characteristics (parity, fetal presentation)

*p < 0.05; **p < 0.01; ***p < 0.001; Results in bold indicate a statistically significant result

IVF, in vitro fertilization; PSM, propensity score matched sample; RR, Risk ratio

Delivery complications include uterine rapture, uterine atony, placental abruption, and immediate postpartum hemorrhage

Finally, the association between MAR and cesarean delivery remained significant even when we additionally adjusted for the history of previous cesarean deliveries (aRR = 1.26, 95% CI = 1.15–1.37 in fully adjusted model and RR = 1.12, 95% CI = 1.02–1.22 in a PSM sample) (see Tables 2 and 3 in the Supplement). Additional analyses also indicated that the observed associations between MAR/IVF and delivery complications were largely driven by an increased risk of uterine-related delivery complications and immediate postpartum hemorrhage (see Tables 3, 4 and 5 in the Supplement).

## Discussion

In this retrospective cohort study of most recent medical records data (2016–2022) from a tertiary hospital in South-central Louisiana, we found that MAR was associated with increased risk for cesarean delivery and delivery complications (e.g., uterine rapture, uterine atony, placental abruption, and immediate postpartum hemorrhage), but not other perinatal and cardiometabolic outcomes included in the analysis. In a propensity score matched sample, the risk of delivery complications was attenuated, suggesting that some of the risk between MAR and delivery complications is explained by maternal factors rather than the treatment. The increased risk of cesarean delivery and delivery complications remained elevated in a propensity-score matched sample limited to singleton and nulliparous deliveries, and additionally controlling for the history of cesarean deliveries. Furthermore, increased risk for cesarean delivery and delivery complications were found in a sample limited to IVF-treated population, and these associations were larger in magnitude compared to MAR (IVF and IUI). This suggests that factors associated with a more invasive MAR treatment, i.e., IVF, may contribute to an increased risk of cesarean delivery and delivery complications.

This study adds to the growing evidence demonstrating higher incidence of cesarean deliveries in pregnancies conceived with MAR [5, 10, 25–27] which, in part, may be attributed to pregnancy complications associated with higher incidence of underlying medical complications (e.g., diabetes, high blood pressure) experienced by the MAR-seeking population [25]. As suggested by previous research [5, 25, 28], underlying disease and health complications rather than fertility treatment appear to be driving the use of cesarean deliveries. However, we found an independent association between cesarean deliveries and MAR even after adjusting for a number of pregnancy risk factors and preexisting health conditions, and additionally controlling for a history of cesarean delivery. Increased rates of cesarean deliveries among MAR-treated population may be also explained by a more specialized obstetric care received by this group and the choice of MAR-patients and their physicians for obstetric interventions (such as labor induction or cesarean delivery) [27]. A better understanding of the underlying reasons and mechanisms between fertility treatment and cesarean deliveries is needed to improve preventive strategies and reduce the possible harm of unnecessary cesarean deliveries, particularly among the nulliparous MAR-treated population with minimal health risks.

Our findings also indicate that women who conceived through IVF are at a higher risk of delivery complications, including placental abruption, uterine rapture, and immediate postpartum hemorrhage. These findings contribute to an increasing body of research that has documented associations between IVF and placental-related complications [29, 30], and severe postpartum hemorrhage [20, 31]. Furthermore, our additional analyses showed that the observed associations between IVF and delivery complications were largely driven by an increased risk of uterine-related delivery complications and immediate postpartum hemorrhage. Further research should investigate which specific components of IVF (e.g., the dose and type of ovarian stimulation drugs, fresh vs. frozen embryo transfer) are related to adverse obstetric outcomes. Furthermore, little research has investigated the impact of IVF on obstetric and perinatal outcomes among certain populations, including those with a diagnosis of infertility, single women, and sexual minority women.

While we did not observe associations between MAR and cardiometabolic complications during pregnancy and childbirth, these findings should be interpreted in the light of recent evidence that reported increased risks of hypertensive and cardiometabolic disorders of pregnancy in MAR-treated populations [2, 17, 20, 21]. In the first population-based study and the largest analysis to consider both obstetric outcomes and vascular complications at time of delivery in women who conceived with MAR, Wu et al. (2022) found that pregnancies conceived with ART were independently associated with increased risk for several vascular complications, even after adjusting for baseline risk profile [2]. Similarly, in their analysis of 46 million delivery hospitalizations in the US from 2008 to 2019, Zahid et al. (2023) found that ART-conceived pregnancies had an increased risk of cardiovascular complications during delivery hospitalizations including preeclampsia/eclampsia, heart failure, cardiac arrhythmias, hemorrhagic stroke, pulmonary edema [21]. However, another recent study found no long-term cardiovascular disease impacts among ART-treated populations compared with those who conceived without fertility treatment [32]. More research is needed to examine short- and long-term effects of different fertility treatments as well as to disentangle the impact of infertility treatments, preexisting comorbidities, and the combination of both.

There are several limitations to be noted. We used delivery hospitalization records from one state in the Southeastern region of the US, and limited generalizations can be made regarding our findings. Further studies are needed to replicate our findings in other states with similarly diverse samples. The generalizability of our results is also limited to the in-hospital delivery population and not to those who used MAR but did not conceive. Studies with larger MAR-treated samples are needed, as a lack of associations between MAR and other outcomes in this study may be due to relatively small samples of MAR pregnancies. Additionally, while we adjusted for a number of potential confounders, we cannot exclude the possibility of residual confounding (e.g., a number of prenatal care visits, diagnosis of PCOS or endometriosis).

Our measures of MAR relied on medical health records and thus we were unable to determine infertility type (e.g., social vs. physiological) and whether the infertility diagnosis was due to female infertility related causes (e.g., PCOS, ovulatory disfunction, tubal disease). Further studies with information on infertility diagnoses and the causes of infertility are critical in determining whether the risk factors associated with infertility diagnoses (versus infertility treatment) are the key drivers in contributing to adverse outcomes in MAR-conceived pregnancies. We also acknowledge potential misclassification of exposure measures, ascertainment bias, and underreporting of study outcomes, all inherent to retrospective study designs [2]. Finally, severity of infertility and the different treatment protocol were also not captured in our study yet may potentially contribute to observed differences in some study outcomes [20].

## Conclusion

In a propensity score matched sample, women who conceived with MAR were at increased risk of cesarean deliveries, whereas IVF was associated with higher risk of cesarean delivery and delivery complications. Overall, these findings suggest the need to identify and implement early preventive and surveillance strategies for women who are at risk and conceive through IVF. Further studies are required to identify treatment-specific risk factors and determine short- and long-risk effects of different fertility treatments.

### Electronic supplementary material

Below is the link to the electronic supplementary material.

Supplementary Material 1
