# Supplementary material for: The risk of perinatal and cardiometabolic complications in pregnancies conceived by medically assisted reproduction
Source: J Assist Reprod Genet. 2024 Jan 20;41(3):613–21. doi: 10.1007/s10815-024-03025-9 (PMC10957823; doi:10.1007/s10815-024-03025-9)
Supplement: Supplementary file 1 — Supplementary Material 1 [file 10815_2024_3025_MOESM1_ESM.docx]

# Table 1: ICD-10 diagnosis codes for study outcomes

| Study outcomes | ICD-10 |
| --- | --- |
| *Cesarean delivery* | n/a |
| *Gestational diabetes mellitus* | O24.410, O24.414, O24.415, O24.419, O24.420, O24.424, O24.425, O24.429,  O24.430, O24.434, O24.435, O24.439, O99.810, O99.814, O99.815 |
| *Hypertensive disorders of pregnancy (gestational hypertension, preeclampsia with or without severe features, eclampsia, or HELLP syndrome)* | O10.011, O10.012, O10.013, O10.019, O10.02, O10.03 O10.211, O10.212, O10.213, O10.219, O10.22, 010.23  O10.311, O10.312, O10.313, O10.319, O10.32, O10.33 O10.411, O10.412, O10.413, O10.419, O10.42, O10.43  O10.911, O10.912, O10.913, O10.919, O10.92, O10.93  O13.1, O13.2, O13.3, O13.4, O13.5, O13.9  O14.00, O14.02, O14.03, O14.04, O14.05  O14.10, O14.12, O14.13, O14.14, O14.15  O14.20, O14.22, O14.23, O14.24, O14.25  O14.90, O14.92, O14.93, O14.94, O14.95 |
| *Delivery complications (uterine rapture, uterine atony, placental abruption, and immediate postpartum hemorrhage)* | O45.001, O45.002, O45.003, O45.009, O45.011, O45.012, O45.013, O45.019, O45.021, O45.022, O45.023, O45.029, O45.091, O45.092, O45.093, O45.099, O45.8X1, O45.8X2, O45.8X3, O45.8X9, O45.90, O45.91, O45.92, O45.93, O62.2, O72.1, O75.89, O71.2, O71.00, O71.02, O71.03, O71.1, O90.0 |
| *Postpartum readmission* | n/a |

HELLP, Hemolysis, Elevated Liver enzymes and Low Platelets syndrome

ICD-10, The International Classification of Diseases

**Table 2**. Pregnancy and delivery outcomes for MAR- and unassisted pregnancies: unmatched (N=57,354) and propensity-score matched (N=2,311) samples additionally controlling for the history of cesarean deliveries

|  | Unmatched sample | | Propensity score-matched sample |
| --- | --- | --- | --- |
|  | Model 1  Crude RR (95% CI) | Model 2  Adjusted RR (95% CI) | Model 3  PSM RR (95% CI) |
| *Cesarean delivery* | **1.62 (1.49-1.76)***** | **1.23 (1.13-1.34)***** | **1.13 (1.03-1.42)**** |
| *Gestational diabetes mellitus* | **1.41 (1.13-1.76)**** | 1.00 (0.81-1.25) | 0.92 (0.72-1.17) |
| *Hypertensive disorders of pregnancy* | **1.33 (1.15-1.55)***** | 1.07 (0.94-1.22) | 1.13 (0.95-1.35) |
| *Delivery complications* | **1.60 (1.22-2.09)***** | **1.46 (1.11-1.93)**** | 1.35 (0.97-1.87) |
| *Postpartum readmission* | 1.57 (0.95-2.60) | 1.25 (0.77-2.04) | 1.21 (0.70-2.09) |

Model 2 adjusted for maternal sociodemographic (age at delivery, race/ethnicity, educational attainment, marital status, insurance), preexisting health conditions (obesity, substance use, chronic hypertension, diabetes mellitus, pulmonary disease, mood and anxiety disorders, history of sexually transmitted infections (STI)), and index pregnancy characteristics (parity, multiple gestation, fetal presentation).

*p<0.05; **p<0.01; ***p<0.001; Results in bold indicate a statistically significant result.

MAR, medically assisted reproduction; PSM, propensity score matched sample; RR, Risk ratio.

Delivery complications include uterine rapture, uterine atony, placental abruption, and immediate postpartum hemorrhage.

**Table 3**. Pregnancy and delivery outcomes for IVF and unassisted pregnancies: unmatched (N= 57,354) and propensity-score matched (N=1,867, IVF n=472, non-IVF n=1,395) samples additionally controlling for the history of cesarean deliveries

|  | Unmatched sample  (N= 57,354) | | Propensity score-matched sample (N=1,395) |
| --- | --- | --- | --- |
|  | Model 1  Crude RR (95% CI) | Model 2  Adjusted RR (95% CI) | Model 3  PSM RR (95% CI) |
| *Cesarean delivery* | **1.62 (1.47-1.78)***** | **1.22 (1.11-1.35)***** | **1.15 (1.03-1.28)*** |
| *Gestational diabetes mellitus* | 1.26 (0.98-1.62) | 0.89 (0.69 -1.14) | 0.76 (0.58-1.01) |
| *Hypertensive disorders of pregnancy* | **1.33 (1.12-1.57)***** | 1.10 (0.95-1.27) | 1.13 (0.93-1.37) |
| *Delivery complications* | **1.92 (1.46-2.51)***** | **1.77 (1.34-2.35)***** | **1.44 (1.04-2.01)**** |
| *Postpartum readmission* | **1.79 (1.07-3.00)*** | 1.42 (0.86-2.36) | 1.33 (0.75-2.34) |

Model 2 adjusted for maternal sociodemographic (age at delivery, race/ethnicity, educational attainment, marital status, insurance), preexisting health conditions (obesity, substance use, chronic hypertension, diabetes mellitus, pulmonary disease, mood and anxiety disorders, history of sexually transmitted infections (STI)), and index pregnancy characteristics (parity, multiple gestation, fetal presentation).

*p<0.05; **p<0.01; ***p<0.001; Results in bold indicate a statistically significant result.

IVF, in vitro fertilization; PSM, propensity score matched sample; RR, Risk ratio.

Delivery complications include uterine rapture, uterine atony, placental abruption, and immediate postpartum hemorrhage.

**Table 4**. Delivery complications for MAR-treated and unassisted pregnancies: unmatched (N=57,354) and propensity-score matched (N=2,311) samples

|  | Unmatched sample  (N= 57,354) | | Propensity score-matched sample (N=2,311) |
| --- | --- | --- | --- |
|  | Model 1  Crude RR (95% CI) | Model 2  Adjusted RR (95% CI) | Model 3  PSM RR (95% CI) |
| *Placenta-related delivery complications (e.g., placental abruption)* | 0.41 (0.06-2.89) | 0.47 (0.06-3.42) | 0.92 (0.05-3.78) |
| *Uterine-related delivery complications (e.g., uterine rupture, uterine atony)* | **3.24 (2.05-5.12)***** | **2.65 (1.66-4.25)***** | 1.87 (0.87-4.01) |
| *Postpartum complications (e.g., immediate postpartum hemorrhage)* | **1.71 (1.10-2.65)*** | **1.63 (1.03-2.57)*** | **1.99 (1.09-3.63)*** |

Model 2 adjusted for maternal sociodemographic (age at delivery, race/ethnicity, educational attainment, marital status, insurance), preexisting health conditions (obesity, substance use, chronic hypertension, diabetes mellitus, pulmonary disease, mood and anxiety disorders, history of sexually transmitted infections (STI)), and index pregnancy characteristics (parity, multiple gestation, fetal presentation).

*p<0.05; **p<0.01; ***p<0.001; Results in bold indicate a statistically significant result.

MAR, medically assisted reproduction; PSM, propensity score matched sample; RR, Risk ratio.

Delivery complications include uterine rapture, uterine atony, placental abruption, and immediate postpartum hemorrhage.

**Table 5**. Delivery complications for IVF and unassisted pregnancies: unmatched (N= 57,354) and propensity-score matched (N=1,867, IVF n=472, non-IVF n=1,395) samples

|  | Unmatched sample  (N= 57,354) | | Propensity score-matched sample (N=2,311) |
| --- | --- | --- | --- |
|  | Model 1  Crude RR (95% CI) | Model 2  Adjusted RR (95% CI) | Model 3  PSM RR (95% CI) |
| *Placenta-related delivery complications (e.g., placental abruption)* | 0.50 (0.07-3.58) | 0.59 (0.08-4.25) | 0.54 (0.07-4.85) |
| *Uterine-related delivery complications (e.g., uterine rupture, uterine atony)* | **4.01 (2.55-6.32)***** | **3.34 (2.10-5.32)***** | **2.55 (1.27-5.12)**** |
| *Postpartum complications (e.g., immediate postpartum hemorrhage)* | **2.03 (1.29-3.18)**** | **1.95 (1.22-3.10)**** | **2.50 (1.41-4.43)**** |

Model 2 adjusted for maternal sociodemographic (age at delivery, race/ethnicity, educational attainment, marital status, insurance), preexisting health conditions (obesity, substance use, chronic hypertension, diabetes mellitus, pulmonary disease, mood and anxiety disorders, history of sexually transmitted infections (STI)), and index pregnancy characteristics (parity, multiple gestation, fetal presentation).

*p<0.05; **p<0.01; ***p<0.001; Results in bold indicate a statistically significant result.

IVF, in vitro fertilization; PSM, propensity score matched sample; RR, Risk ratio.

Delivery complications include uterine rapture, uterine atony, placental abruption, and immediate postpartum hemorrhage.
